# Supplementary material for: Computer vision supports primary access to meat by early Homo 1.84 million years ago
Source: PeerJ. 2022 Oct 18;10:e14148. doi: 10.7717/peerj.14148 (PMC9586113; doi:10.7717/peerj.14148)
Supplement: Supplemental Information 1 [file peerj-10-14148-s001.docx]

**Supplementary Information**

**Computer vision indicates primary access to meat by early Homo 1.84 million years ago**

Lucía Cobo-Sánchez^1,2^, Marcos Pizarro-Monzo^2^, Gabriel Cifuentes-Alcobendas^2,7^, Blanca Jiménez-García^2,3^, Natalia Abellán^2,3^, Lloyd A. Courtenay^4^, Audax Mabulla^5^, Enrique Baquedano^2,6^, Manuel Domínguez-Rodrigo^2,7,8^ *

^1^Computational Archaeology (CoDArchLab), Institute of Archaeology, University of Cologne, Albertus-Magnus-Platz, D-50923, Cologne, Germany

^2^Institute of Evolution in Africa (IDEA), Alcalá University and Archaeological and Paleontological Museum of the Community of Madrid, Covarrubias 36, 28010 Madrid, Spain.

^3^Department of Artificial Intelligence of UNED (National University for Distance Education) 28040 Madrid, Spain

^4^Department of Cartographic and Terrain Engineering, Superior Polytechnic School of Ávila, University of Salamanca, Spain.

^5^Department of Archaeology and Heritage Studies, University of Dar es Salaam, P.O. Box 5050, Dar es Salaam, Tanzania.

^6^ Archaeological andPaleontological Museum of the Community of Madrid, Plaza de las Bernardas s/n, Alcalá de Henares, Spain.

^7^Area of Prehistory (Department History and Philosophy), University of Alcalá, 28801 Alcalá de Henares, Spain.

^8^Department of Anthropology, Rice University, 6100 Main St., Houston, TX 77005-1827

* Corresponding author

**INDEX**

Background on East African Early Pleistocene Archaeology and hominin behavior ……………. 2

David’s Site (DS) …………………………………………………………………………………. 3

Stratigraphic and geoarchaeological overview of DS …..…………………………………............ 4

Taphonomic approaches to reconstruct site formation of early Pleistocene faunal assemblages … 6

Reconstruction of the taphonomic history at the DS 22B assemblage ……………………………. 8

References ………………………………………………………………………………………… 22

Major indicators of the experimental transfer models …………………………………………… 27

**1. Background on East African Early Pleistocene Archaeology and hominin behavior.**

Most of what is known about the subsistence behavior of early *Homo* comes from several sites at Olduvai Gorge (Tanzania), Kanjera South (Kenya), and Koobi Fora (Kenya) that are dated to 2.0 - 1.6 Ma[^1–6^](https://paperpile.com/c/LwZzJO/13RPD+kjugH+YZXBM+tWRZD+coMLZ+o8suK). At Olduvai Gorge, archaeological remains have been preserved mostly in undisturbed contexts, which has promoted abundant archaeological excavations and discussions about early human behavior during the past decades[^1^](https://paperpile.com/c/LwZzJO/13RPD). FLK *Zinj* has constituted the most informative site at Olduvai for decades, since it was the only known anthropogenic site from Bed I. All the other Bed I sites represented non-anthropogenic accumulations, including carnivore-collected carcasses, background scatters or palimpsests in which hominins contributed only marginally to the bone assemblages[^1^](https://paperpile.com/c/LwZzJO/13RPD).

The FLK *Zinj* site has been studied by many researchers and from many different viewpoints, and a wealth of detailed information exists about its formation and autochthonous nature, the contribution of hominins and carnivores to the archaeofaunal assemblage, the inferred carcass foraging strategies of hominins, the characteristics of the surrounding environment, and the spatial properties of the distribution of the remains[^1,7–15^](https://paperpile.com/c/LwZzJO/GZxk8+iR84r+hfXu8+13RPD+AUl7P+IhOAc+UUlPm+SHJfp+0qlkX+7FqA5). Although there is a long history of controversial interpretations of the FLK Zinj site (see reviews in [^16^](https://paperpile.com/c/LwZzJO/hYUL)^;^[^17^](https://paperpile.com/c/LwZzJO/R95x); [^18^](https://paperpile.com/c/LwZzJO/5kAL); [^19^](https://paperpile.com/c/LwZzJO/mfgj), most taphonomic studies have concluded that hominins were actively accumulating carcasses at FLK *Zinj*, which they probably acquired through hunting or confrontational scavenging from felids[^1,7,11,20,21^](https://paperpile.com/c/LwZzJO/EFcHa+GZxk8+13RPD+IhOAc+7QhYP).

Recent discoveries at Olduvai are reinforcing our impression that early Pleistocene hominins had the cognitive capacity needed to engage in collective and collaborative activities and a socially more complex behavior than previously thought. Three new large anthropogenic sites discovered by TOPPP (the Olduvai Paleoanthropology and Paleoecology Project) in recent years suggest that hominins were actively and regularly obtaining abundant meat resources. PTK (Philip Tobias Korongo), DS (David’s Site), and AGS (Alberto Gómez’s Site) lie on the same paleosurface as FLK *Zinj*, which means that this site can cease to be considered an anomaly in the Olduvai early Pleistocene archaeological record. Furthermore, the discovery in 2015 at PTK of OH 86, a phalanx of a modern human-like hand, has proven that a hominin with a more modern-looking anatomy coexisted with *Homo habilis* and *Paranthropus boisei* in Olduvai during Bed I times [^22^](https://paperpile.com/c/LwZzJO/jvrps). This phalanx could have belonged to *Homo ergaster* or to its more ancient form.

Within the last decades, archaeological research in other regions outside of Africa has demonstrated that the earliest evidence of hunting dates to much earlier times than previously thought. It certainly predates the appearance of anatomically modern humans, and flexible hunting and scavenging strategies probably characterized foraging among different hominin populations and species of the genus *Homo* throughout its evolution[^23–28^](https://paperpile.com/c/LwZzJO/FB08M+9PBlN+XjUrA+V6Mvf+itEZc+Hc76i). Regarding the zooarchaeological and taphonomic data from 1.8 – 1.6 Mya, the evidence indicates that hominins were already gaining access to complete carcasses of relatively large ungulates regularly, and repeatedly transporting large portions of these carcasses back to favored safer locations, as once proposed by [^29^](https://paperpile.com/c/LwZzJO/DuBIG)^,^[^30,31^](https://paperpile.com/c/LwZzJO/uRWyW+pmrXP). In spite of the disagreements between researchers regarding the earliest foraging strategies of hominins, most authors acknowledge that the appearance of some early *Homo* was marked by an increase in body size and cranial capacity, and the emergence of modern gut morphology among other changes, brought about by a dietary shift toward a high-quality diet focused on the consumption of meat[^32^](https://paperpile.com/c/LwZzJO/JxP1V).

**2. David’s Site (DS)**

DS (1.84 Ma) is located in the junction between the main and secondary branches of Olduvai Gorge in northern Tanzania (2º59’33’’S; 35º21’08’’E). It was discovered a few hundred meters away from FLK *Zinj* and the archaeological deposit lay very close to the surface [^33^](https://paperpile.com/c/LwZzJO/eBRCD). From 2014 to 2018 intensive excavations exposed an area of 554 m^2^. DS is larger than FLK *Zinj* site and constitutes the biggest open window to the African Early Pleistocene (**Figure S1**). Over the course of five fieldwork seasons, more than 15,000 fossil remains and stone tools (including sieve finds) were recovered from Level 22B. The overlying Level 22A contained archaeological remains as well, yet in much lower densities. Careful excavation of the layers by a large digging crew included plotting the materials larger than 2 cm with laser theodolites. All trenches (3x3 m) were stereo-photographed once they were uncovered in order to obtain a photogrammetric 3D reconstruction of the soil and deposited materials as they were prior to recovery. Azimuth and plunge of the items were measured with a compass and an inclinometer respectively. The removed sediment was sieved in 5 mm and 3 mm meshes.

There are several aspects that bestow a special value on DS. On one side, preservation of the fossil bones is as good as that at FLK *Zinj*, and reflects that post-depositional processes had a very marginal effect on the assemblage. Also, numerous rib and vertebrae remains have been recovered that usually disappear due to the action of carnivores or hydraulic processes. On the other side, the assemblage was probably formed in a short period of time[^15^](https://paperpile.com/c/LwZzJO/7FqA5) and the ecological conditions at the site can be reconstructed with very high resolution, not to mention that given its large extension, all aspects, taphonomic, behavioral and ecological, can be explored spatially. In fact, recent work pertaining to the lithic assemblage of DS also explores the spatial magnitude of the technological, in an effort to reconstruct hominin spatial behavior [^34^](https://paperpile.com/c/LwZzJO/HBUW). The results from the biochemical analysis of plant remains and the geoarchaeological studies will soon appear and complement the results presented here.

**3. Stratigraphic and geoarchaeological overview of DS**

From a geological point of view, DS lies in Bed I, which spans from approximately 1.98 Ma to 1.75 Ma, and is formed by the alternating deposition of clayey facies and volcanic tuffs (1A – 1F) that have been securely dated[^35–38^](https://paperpile.com/c/LwZzJO/a2TUi+r2qlF+Npi4z+XpQab). The paleosol underlying Tuff 1C, which contains the archaeological deposit, is a clay stratum (<20 cm) that can be traced laterally on a significant portion of the gorge at the junction and on both ends of its trajectory in areas that lay close to an ancient lake. Paleoecological reconstructions of the *Zinj* paleolandscape have emphasized the low-energy depositional environment dominating the lower and middle sequence of Bed I around FLK *Zinj* (and the other anthropogenic sites)[^31^](https://paperpile.com/c/LwZzJO/pmrXP).

The paleolandscape was composed of a raised platform and a shallow depression separated by around 2 m difference in elevation (Ashley et al., 2010; Uribelarrea *et al.*, 2014). FLK *Zinj*, PTK, AGS, and DS are all located on topographically higher ground in a supralittoral environment and are separated from each other by a few hundred meters[^31^](https://paperpile.com/c/LwZzJO/pmrXP); **Figure S1**). DS in particular is located between two alluvial inputs. Mineralogical and geo­chemical lateral variations throughout the FLK *Zinj* paleolandscape suggest a differential entry of fresh water into the basin, with fresh water entering the system from the surroundings of DS during the deposition of both levels 22A and 22B[^39^](https://paperpile.com/c/LwZzJO/8IgC0). This suggests that at DS hominins probably had regular access to fresh water and herbivores. Phytolith analyses have shown that like FLK *Zinj* and PTK, DS was located in a wooded environment, which could have provided refuge from carnivore predation [^40^](https://paperpile.com/c/LwZzJO/QLEcL).

Level 22B is deposited disconformably over an irregular topography of the Chapati Tuff [^31^](https://paperpile.com/c/LwZzJO/pmrXP)), a laminated reworked tuff present throughout the entirety of the Main Junction. In most parts of the site Level 22B overlies the Chapati Tuff with a discrete and sharp contact[^41^](https://paperpile.com/c/LwZzJO/MsdIO); [^31^](https://paperpile.com/c/LwZzJO/pmrXP); [^39^](https://paperpile.com/c/LwZzJO/8IgC0)). Archaeological remains in level 22B are commonly found in the lowermost ~5 cm. This layer was deposited under very low energy conditions[^39^](https://paperpile.com/c/LwZzJO/8IgC0). An intercalated, discontinuous <5 cm silty unit, Level 22 Silt, is found between levels 22A and B at DS and surroundings[^31^](https://paperpile.com/c/LwZzJO/pmrXP)^,^[^39^](https://paperpile.com/c/LwZzJO/8IgC0) but does not seem to have altered the position of the archaeological remains in a meaningful way. Level 22A is a ~10 cm earthy clay, discontinuous, low energy deposit[^39^](https://paperpile.com/c/LwZzJO/8IgC0), which overlies level 22 Silt in the northern areas of the site, is commonly absent in the center of the site, but overlies level 22B in the southern grids of the site. Archaeological remains embedded in this level are also found in the lowermost ~5 cm of the level. Tuff 1C deposited conformably over level 22A and 22silt[^41^](https://paperpile.com/c/LwZzJO/MsdIO)^,^[^31^](https://paperpile.com/c/LwZzJO/pmrXP) (**Figure S1**).


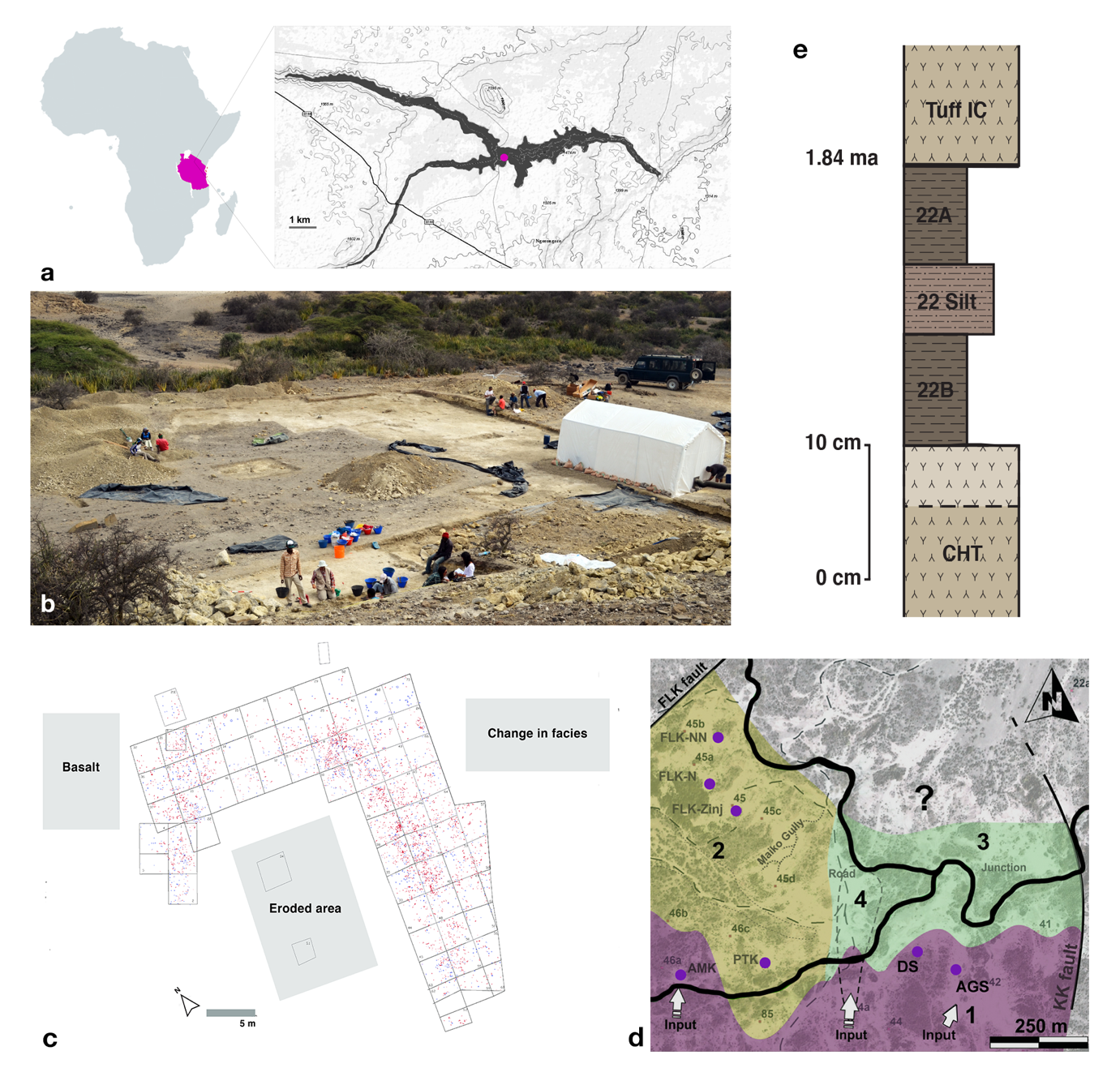


**Figure S1.** **David’s Site (DS)***.* **a,** Location of DS in Olduvai Gorge in northern Tanzania. **b,** General view of the DS excavation in 2015 from the south. **c,** Site plan of the excavation of level 22B at DS showing the distribution of the recovered archaeological materials > 20 mm. The site is delimited by erosion, ancient lava flows and a change in facies. In the central area of the site, the deposition of the Ndutu unconformable sediments and their subsequent erosion had exposed the clay deposit to the effect of rains and the use of the area as a road. In addition, the southern edge of the excavation had started yielding lower densities of materials due to a change in facies from clayey to silty sediments, which could be indicating a shift in the paleolandscape. The higher presence of large carbonate nodules could have affected the preservation of bone remains at the south-eastern limit of the site, where fossils are almost completely absent and where only large basalts and some stone tools have been recovered. Lava flows that were already part of the paleolandscape when hominins created the site delimit the remaining areas of the site. **d,** Cartography of the main zones identified in the *Zinj* paleolandscape, within the lake-margin zone of lower-middle Bed I between the FLK and KK faults and the location of the main Bed I sites, including DS (modified from Figure 10 in [^31^](https://paperpile.com/c/LwZzJO/pmrXP). The anthropogenic sites are located on zones 1 and 2, which are the topographically highest areas on the landscape. **e,** General stratigraphic section between the Chapati Tuff (CHT) and Tuff IC at DS. (Modified from Figure 2 in[^39^](https://paperpile.com/c/LwZzJO/8IgC0)).

**4. Taphonomic approaches to reconstruct site formation of early Pleistocene faunal assemblages**

Three of the major issues regarding hominin lifestyles with which researchers have been concerned in the past fifty years of archaeological research in East Africa include the identification of the main agents of site formation, the hunting or scavenging behavior of hominins reflected at the sites, and the socioeconomic function of these locations. Importantly, the argumentation in these debates has been of a taphonomic nature, as these topics have centered around the questions of whether skeletal part representation, bone surface modifications, and bone breakage patterns at the sites, particularly at FLK *Zinj*, reflected hominin or carnivore agency, and primary or secondary access to the carcasses by hominins. This has prompted a significant expansion of taphonomic approaches in the past decades.

Nowadays, taphonomic research counts with a considerable number of robust methods that allow the scientific contrasting of hypotheses and assumptions. Experimental and replicative studies serve to generate comparative taphonomic data and play a key role in the analyses of early sites. They model time and sequence of carcass acquisition by hominins and non-human carnivores and the intensity of carcass processing resulting from different scenarios [^28^](https://paperpile.com/c/LwZzJO/Hc76i). Experimental work has done much to eliminate equifinality from taphonomic interpretations. General differences detected in fracture angles created through dynamic and static loading [^42,43^](https://paperpile.com/c/LwZzJO/AtbUA+0wvFA), contrasts in the frequencies of tooth-marked shaft fragments depending on the timing of access to carcasses by carnivores [^44–46^](https://paperpile.com/c/LwZzJO/PPrE0+wmESE+TS4nH) or in the frequencies of cut-marked specimens in scenarios of primary and secondary access to carcasses by hominins [^47,48^](https://paperpile.com/c/LwZzJO/drLUh+g7uOG) have been useful to support different models of access to carcasses by hominins.

Yet, on occasions, the application of experimental data to the archaeological record has generated inconsistent results depending on the analyst, like in the case of FLK *Zinj*. This is in part due to differences in how researchers quantify different taphonomic attributes, but it is also related to problems of equifinality often caused by small experimental samples and to the examination of one single variable at a time.

Domínguez-Rodrigo et al. [^49–51^](https://paperpile.com/c/LwZzJO/FuLXo+ttAJq+GIdfG) have advocated the combination of variables in multivariate statistical approaches in order to overcome equifinality produced when variables are used independently. When variables are used jointly in multivariate analyses, the results are much more consistent, because the relationships and associations between variable types are captured in addition to the effects of each single variable [^49–51^](https://paperpile.com/c/LwZzJO/FuLXo+ttAJq+GIdfG). Several studies combining the distributions of all mark types (tooth marks, cut marks and percussion marks on different anatomical portions) or different variables related to skeletal part profiles, have widely demonstrated that viewing assemblage formation as a dynamic system in which the association of taphonomic entities generates emergent properties is far more effective [^49–52^](https://paperpile.com/c/LwZzJO/FuLXo+ttAJq+GIdfG+r9UKs). Recently, the application of machine learning algorithms to taphonomic research has been shown to have the power to magnify the advantages of using many variables simultaneously. These methods are much more powerful than traditional frequentist and Bayesian statistical methods. A set of several machine learning algorithms, including Support Vector Machines, Neural Networks, and Random Forests, have been applied in recent years with high success to various areas of taphonomic research like skeletal part profiles, bone surface modifications and breakage patterns, yielding a strong convergence in the classifications (sometimes with 100% accuracy) [^52–55^](https://paperpile.com/c/LwZzJO/r9UKs+CVqrW+OkkXV+BNkFv).

The taphonomic analyses included in this supplementary section were carried out to reconstruct the formation of the faunal assemblage from Level 22B at DS and examine the effect of the abiotic and biotic taphonomic agents involved in the creation and transformation of the original archaeological deposit. The goal is to accurately determine the degree of im­plication of hominins at the site, and the kind of interaction that took place between them and the carnivores with which they coexisted.

The analysis includes traditional taphonomic methods and groundbreaking approaches, that range from univariate and bivariate statistical methods as well as multivariate approaches and machine learning methods. The latter really contribute to eliminate equifinality in every analysis. The application of these methods also includes the use of the available experimental data modeling different types of access to carcasses by hominins and carnivores, as well as actualistic studies carried out in the wild of modern carnivore ethology as referential frameworks.

We build on these previous taphonomic analyses of Bed I sites in Olduvai Gorge and examine site integrity, by using a combination of approaches that include bone orientation patterns, specimen size distribution and a multivariate analysis of the frequencies of different types of bone shapes and composition. The goal of this analysis is mainly to test the hypothesis that the site is autochthonous and that the effect of water currents on the archaeological accumulation can be excluded, and thereby that the spatial properties of the assemblage are intact. Then, we analyze skeletal part abundances and their relationship to food utility and return rates. The included analyses of bone fragmentation patterns and bone surface modifications are especially useful to assess time of access to carcasses by hominins and carnivores. Elucidating whether hominins had early and primary access to carcasses is necessary to justify food surplus and intentional food sharing. If primary access to carcasses is confirmed then abundant meat and butchery, active foraging strategies, food transport, food sharing and cooperation are more likely, since primary access is a prerequisite for these behaviors.

Access to carcasses by hominins is addressed additionally by inspecting the anatomical location of cut marks aside from their frequencies, and site agency can also be addressed through the taphotype approach, which serves to classify assemblages as hominin or carnivore-made according to predominant patterns of long bone portion deletion [^56^](https://paperpile.com/c/LwZzJO/nvtv4). Following previous analyses of other Bed I sites, we have also included an assessment of the degree of carnivore ravaging at DS, which is useful to infer the degree of carnivore competition present in the surroundings of the site [^1^](https://paperpile.com/c/LwZzJO/13RPD).

**5. Reconstruction of the taphonomic history at the DS 22B assemblage**

This section summarizes additional taphonomic evidence that supports primary access to meat by early *Homo*.

**5.1. Site formation**

The analyses addressing the site’s integrity indicate that DS is exceptionally well preserved, autochthonous and largely undisturbed. The sedimentary matrix in which the DS assemblage was recovered is composed of clay and silty clay, which demonstrates that it was formed in a very low-enery depositional environment. Polished or abraded specimens were very infrequent (less than 0.3%) and this alteration when documented did never affect the entire specimen. Evidence of water-induced and chemical modifications on bone surfaces were rare (less than 0.5%), but around 10% of the fossils showed a carbonate matrix, which 60% of the times hindered the identification of the bone specimen. Subaerial weathering was almost non-existent (99.9% of specimens fall into stage 0, [^57^](https://paperpile.com/c/LwZzJO/227r), which means that the assemblage formed probably in less than a year or just slightly more if a dense vegetation cover existed at the site.

The DS assemblage lacks any evidence indicative of transportation by water flows. The results of the analyses shown in **Figure S2** and **Table S1** show that the assemblage was not affected by significant post-depositional disturbance. Specifically, the completely uniform distribution of the orientation of bones including long bone shafts, and the overwhelming presence of small bone specimens are not consistent with the disturbances created in accumulations by water inputs. Moreover, the similarities in the frequencies of bone specimen composition and shape types between DS and an undisturbed experimental accumulation is further suggestive of completeness and overall integrity of the site. Minimal divergence from the experimental sample is mainly due to the lack of representativeness of cubic or polyhedral bones (mostly vertebrae or compact bones) and, to a lesser extent, of trabecular or cancellous bones (long bone epiphyses and axial elements) in DS as opposed to the Maasai settlement. This difference could however be accounted for by density-mediated attrition and some, although limited, carnivore ravaging. The exceptional preservation of the site also suggests that the spatial properties of the assemblage, as hominins might have left it, might to a great extent be intact.


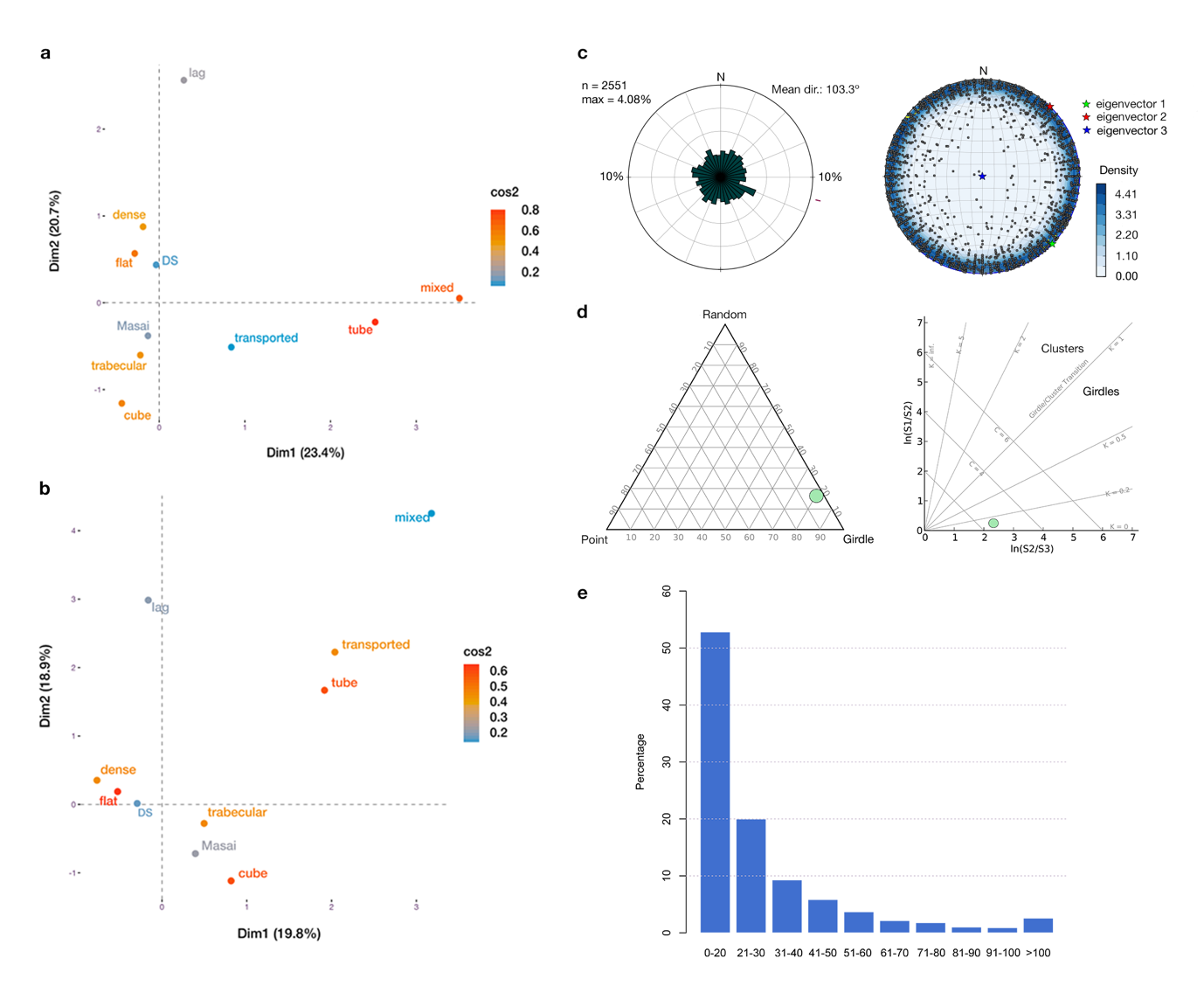


**Figure S2.** **Autochthony and fluvial impact. a,** Bidimensional solution of a multiple correspondence analysis (carried out using the “FactoMineR” library in R [^58^](https://paperpile.com/c/LwZzJO/XTdX) on the sample of small carcasses from DS 22B considering bone shape and bone composition (see [^51^](https://paperpile.com/c/LwZzJO/GIdfG) for a detailed explanation of this approach). The solution explains 44% of inertia. The DS assemblage appears closest to an undisturbed Maasai experimental sample, where trabecular bone fragments are more represented with respect to the other assemblages. The position of DS next to the categories dense and flat suggests that the small carcass sample was not affected by water disturbance. **b,** MCA on the sample of medium-sized carcasses explaining 39% of inertia. DS appears close to the Maasai undisturbed experimental sample. Both are characterized by dense, flat and trabecular bone specimens and compact spongy bones. They appear clearly separated from the lag and transported assemblages. **c,** Rose diagram and stereogram showing a uniform distribution and horizontal trend in the 22B assemblage. Both types of graphs were generated using the software OpenStereo. [^59^](https://paperpile.com/c/LwZzJO/piSZ). **d,** Woodcock and Benn’s diagrams [^59–61^](https://paperpile.com/c/LwZzJO/piSZ+jJrC+cjKc) identifying the DS sample as an isotropic fabric (**Table S1**). **e,** Specimen size distribution of bone remains. The abundance of small fragments indicate minor post-depositional effects by sedimentary processes.

**Table S1.** Circular statistical tests applied to the three DS assemblages (using the “circular” library in R [^62^](https://paperpile.com/c/LwZzJO/SGtw). A: bone and lithics; B: bones; C: long bone shafts. All significance values indicate isotropy.

|  | **Rayleigh** |  | **Kuiper** |  | **Watson** |  | **von Mises d.** | |
| --- | --- | --- | --- | --- | --- | --- | --- | --- |
|  | **Z** | **p-value** | **V** | **p-value** | **U^2^** | **p-value** | **k** | **c** |
| A | 0.0214 | 0.3095 | 15.511 | 0.10<p<0.15 | 0.1003 | >0.10 | 0.00 | 2.11 |
| B | 0.0083 | 0.8543 | 11.569 | >0.15 | 0.0519 | >0.10 | 0.02 | 2.19 |
| C | 0.0182 | 0.7642 | 11.527 | >0.15 | 0.0495 | >0.10 | 0.03 | 2.14 |

**5.2. Skeletal part representation**

The bovid dental remains found at DS 22B belong to *Kobus sigmoidalis*, *Parmularius altidens*, *Connochaetes sp*., *Tragelaphus strepsiceros*, *Megalotragus sp.*, and *Antidorcas recki* (**Figure S3**). Skeletal part profiles of small and medium-sized carcasses present some differences: whereas all skeletal parts are represented similarly in small carcasses, which suggests that they were transported complete to the site, medium-sized carcasses are represented unevenly by anterior and posterior limb bones, with front limbs being more abundant than hind limbs (Figure S3, Tables S2 and S3). Correlations with bone mineral density and food utility and the evenness index did not provide consistent significant results and therefore not a clear answer to the problem of differential representation of skeletal parts at DS. However, some inferences could be drawn from them. Density-mediated attrition affects axial remains especially but is not the only factor that produces the reverse pattern in medium-sized carcasses (Table S4). This means that skeletal profiles of medium-sized carcasses are probably the result of hominin transport decisions. No clear relationship between skeletal part abundances and food utility values was documented either (Table S5). It is likely that the skeletal profile of medium-sized carcasses represents a collection of individual transport episodes that formed under different situations, and that this hinders the observation of any predominant strategy. Since all skeletal elements are represented at the site, it can be assumed that on some occasions all parts of the carcasses were transported to the consumption site, whereas other times hominins only transported selected parts. This bias, which is however not reflected in the evenness index, might have implications regarding transport distance. Whereas the pattern of small carcasses is indicative of short distances to the site, incomplete carcasses point to longer distances or different acquisition strategies. This issue is explored further elsewhere (Cobo-Sánchez et al. in preparation).

**
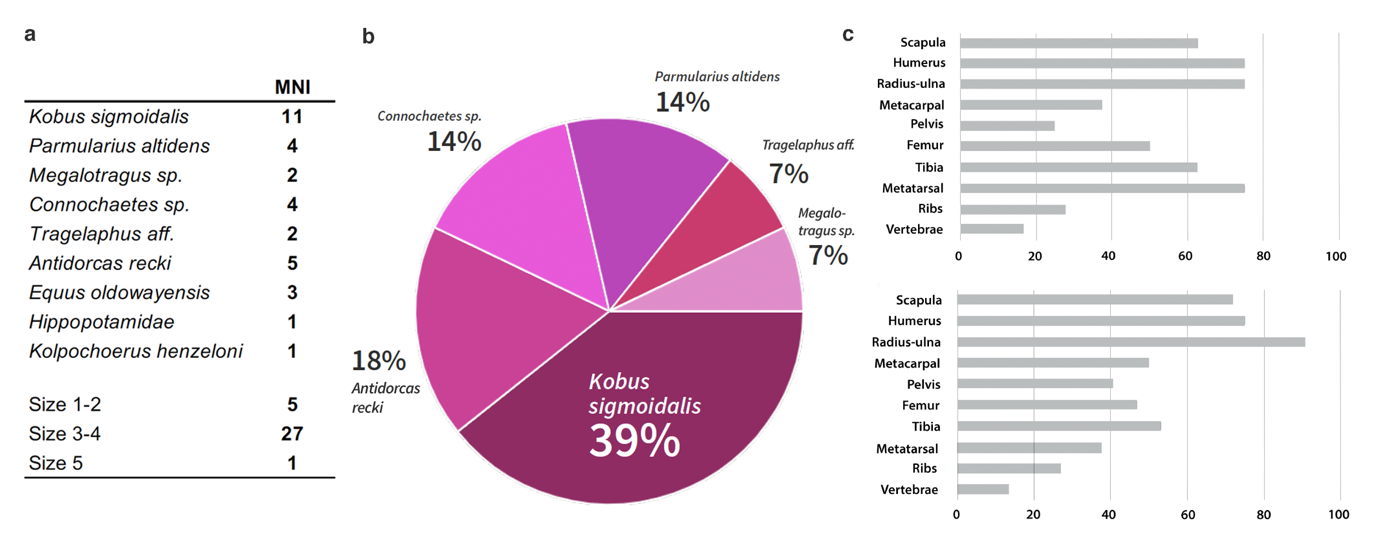
**

**Figure S3. Taxa and skeletal part representation. a,** Minimum number of individuals represented at DS 22B. There are a minimum of 27 individuals that reach size 3-4 (see [^63^](https://paperpile.com/c/LwZzJO/X433e) for definition of animal size classes) in their adult stage, 5 size 1-2 bovids, and 1 size 5 carcass represented that could be identified to species using the dental remains. When considering only limb bones in order to estimate the minimum number of carcasses represented, the radii-ulnae yield a MNI of 4 small carcasses and 16 medium-sized carcasses. **b**, Diversity of bovid taxa representation at DS 22B. Frequencies of represented bovid taxa are similar to those documented at FLK *Zinj* [^1^](https://paperpile.com/c/LwZzJO/13RPD). **c,** Skeletal part profiles of small (top) and medium-sized (bottom) carcasses. Skeletal parts are represented similarly in small carcasses, indicating that they were transported complete to the site (unconstrained strategy). Medium-sized carcasses are represented unevenly by anterior and posterior limb bones, with front limbs being more abundant than hindlimbs. Correlations of skeletal part abundances with bone mineral densities [^64^](https://paperpile.com/c/LwZzJO/zKT5); [^65^](https://paperpile.com/c/LwZzJO/1nQk) show that density-mediated attrition mainly affected the preservation of the axial skeleton. The uneven representation of front and hindlimbs should therefore be explained in terms of hominin carcass acquisition and transportation strategies.

**Table S2**. NISP estimates per element and animal size class of the 22B ungulate assemblage used in the analysis of skeletal part abundances (2014-2016). Appendicular elements are subdivided into proximal, midshaft, and distal portions. Epiphyseal portions with a significant part of midshaft attached were counted twice, once as a shaft and once as an epiphysis.

|  |  | NISP |  |  |  |  |
| --- | --- | --- | --- | --- | --- | --- |
| Element |  | Size 1-2 | Size 3-4 | Size 5 | Size indet | Total |
| Skull |  | 7 | 50 | 1 | 4 | 62 |
| Mandible |  | 9 | 48 | 1 | 1 | 58 |
| Teeth |  | 8 | 46 | 1 | 34 | 89 |
| Vertebrae |  |  |  |  |  |  |
|  | Atlas | 0 | 2 | 0 | 0 | 2 |
|  | Axis | 0 | 1 | 0 | 0 | 1 |
|  | Cervical | 2 | 13 | 0 | 0 | 15 |
|  | Thoracic | 7 | 48 | 0 | 0 | 55 |
|  | Lumbar | 5 | 17 | 0 | 0 | 22 |
|  | Sacra | 0 | 8 | 0 | 0 | 8 |
|  | Caudal | 0 | 9 | 0 | 0 | 9 |
| Ribs |  | 102 | 406 | 2 | 1 | 511 |
| Scapula |  | 7 | 41 | 0 | 0 | 48 |
| Pelvis |  | 11 | 42 | 2 | 4 | 59 |
| Humerus | Complete | 0 | 0 | 0 | 0 | 0 |
|  | Proximal | 1 | 8 | 0 | 0 | 9 |
|  | Midshaft | 25 | 138 | 2 | 8 | 170 |
|  | Distal | 4 | 20 | 0 | 0 | 24 |
| Radius-Ulna | Complete | 0 | 0 | 0 | 0 | 0 |
|  | Proximal | 6 | 36 | 0 | 0 | 42 |
|  | Midshaft | 14 | 70 | 5 | 2 | 91 |
|  | Distal | 2 | 4 | 0 | 0 | 6 |
| Metacarpal | Complete | 0 | 1 | 0 | 0 | 1 |
|  | Proximal | 1 | 16 | 0 | 0 | 17 |
|  | Midshaft | 7 | 34 | 2 | 0 | 43 |
|  | Distal | 2 | 7 | 0 | 0 | 9 |
| Femur | Complete | 0 | 0 | 0 | 0 | 0 |
|  | Proximal | 8 | 7 | 0 | 0 | 15 |
|  | Midshaft | 35 | 67 | 3 | 2 | 107 |
|  | Distal | 1 | 5 | 1 | 0 | 7 |
| Tibia | Complete | 0 | 0 | 0 | 0 | 0 |
|  | Proximal | 1 | 5 | 0 | 0 | 6 |
|  | Midshaft | 35 | 113 | 5 | 2 | 155 |
|  | Distal | 4 | 2 | 0 | 0 | 6 |
| Metatarsal | Complete | 2 | 1 | 0 | 0 | 3 |
|  | Proximal | 4 | 8 | 0 | 0 | 12 |
|  | Midshaft | 12 | 23 | 0 | 0 | 35 |
|  | Distal | 0 | 5 | 0 | 0 | 5 |
| Carpals |  | 4 | 9 | 1 | 0 | 15 |
| Tarsals |  | 4 | 9 | 0 | 0 | 13 |
| Phalanges |  | 15 | 28 | 0 | 0 | 43 |
| Other |  |  |  |  |  |  |
|  | Sesamoid | 0 | 7 | 0 | 0 | 7 |
|  | Patella | 2 | 6 | 0 | 0 | 8 |
|  | Sternum | 0 | 3 | 0 | 0 | 3 |
| Total |  | 347 | 1363 | 23 | 51 | 1790 |

**Table S3**. MNE estimates per element and animal size class of the 22B assemblage used in the analysis of skeletal part abundances (2014-2016).

| Element |  | Size 1-2 | Size 3-4 | Size 5 |
| --- | --- | --- | --- | --- |
| Skull |  | 4 | 10 | 1 |
| Mandible |  | 1 | 21 | 1 |
| Vertebrae |  |  |  |  |
|  | Atlas | 0 | 1 | 0 |
|  | Axis | 0 | 1 | 0 |
|  | Cervical | 1 | 11 | 0 |
|  | Thoracic | 7 | 23 | 0 |
|  | Lumbar | 5 | 15 | 0 |
|  | Sacra | 0 | 5 | 0 |
|  | Caudal | 0 | 9 | 0 |
| Ribs |  | 29 | 112 | 1 |
| Scapula |  | 5 | 23 | 0 |
| Pelvis |  | 2 | 13 | 1 |
| Humerus |  | 6 | 24 | 1 |
| Radius-Ulna |  | 6 | 29 | 3 |
| Metacarpal |  | 3 | 16 | 2 |
| Femur |  | 4 | 15 | 3 |
| Tibia |  | 5 | 17 | 4 |
| Metatarsal |  | 6 | 12 | 0 |
| Carpals |  | 4 | 9 | 1 |
| Tarsals |  | 4 | 9 | 0 |
| Phalanges |  | 14 | 16 | 0 |
| Other |  |  |  |  |
|  | Sesamoids | 0 | 7 | 0 |
|  | Patella | 2 | 6 | 0 |
|  | Sternum | 0 | 1 | 0 |

**Table S4**. Spearman’s rho and p-values of the correlations between bone mineral density and skeletal part abundances of small and medium-sized carcasses (density values after [^64^](https://paperpile.com/c/LwZzJO/zKT5) for medium-sized carcasses and [^65^](https://paperpile.com/c/LwZzJO/1nQk) for small carcasses).

|  | Size 1-2 |  | Size 3-4 |  |
| --- | --- | --- | --- | --- |
|  | Spearman’s rho | p-value | Spearman’s rho | p-value |
| a) |  |  |  |  |
| Complete skeleton | 0.801 | 0.001 | 0.5268 | 0.0436 |
|  |  |  |  |  |
| Bootstrapped data | Mean | p-value | Mean | p-value |
| b) |  |  |  |  |
| Appendicular skeleton | 0.3133 | < 0.0001 | -0.7840 | < 0.0001 |

**Table S5**. Spearman’s rho and p-values of the correlations of skeletal part abundances of small- and medium-sized carcasses with food utility values. The %MAU values of small carcasses are compared against the Modified General Utility Index (MGUI) for sheep (Binford 1978), and the %MAU values of medium-sized carcasses are compared against the Standardized Food Utility Index (SFUI) for caribou (Metcalfe and Jones, 1988).

|  | Size 1-2 |  | Size 3-4 |  |
| --- | --- | --- | --- | --- |
|  | Spearman’s rho | p-value | Spearman’s rho | p-value |
| a) |  |  |  |  |
| Complete skeleton | - 0.4591 | 0.0638 | - 0.2451 | 0.3417 |
| High-survival set | - 0.2927 | 0.4816 | - 0.4048 | 0.3268 |
| Appendicular skeleton | 0.0304 | 0.9545 | - 0.3189 | 0.5379 |
|  |  |  |  |  |
| Bootstrapped sample | Mean | p-value | Mean | p-value |
| b) |  |  |  |  |
| Complete skeleton | - 0.4365 | < 0.0001 | - 0.2422 | < 0.0001 |
| High-survival set | - 0.2479 | < 0.0001 | - 0.3871 | < 0.0001 |
| Appendicular skeleton | 0.0731 | 0.0002 | - 0.2862 | < 0.0001 |

**5.3. Bone breakage patterns and bone surface modifications**

Overall, the bone breakage assessments applied to DS do not clearly point to a single bone breakage agent and suggest that both hominins and hyenas contributed to fracturing at DS (Figure S4; Table S6). Yet, the fragmentation ratios and particularly the machine learning analyses combining bone fracture pattern and notch type distribution (Moclán), point to a scenario in which most of the fracturing (around 90%) was clearly the result of anthropic activities (Figure S4). There is also a high number of impact flakes (83), which are usually attributed to anthropic breakage. Similarly, at FLK *Zinj*, the study of notches and platform angles demonstrated that carnivores were partially involved in bone fracturing, although hammerstone percussion was clearly predominant, especially for large-sized carcasses [^17^](https://paperpile.com/c/LwZzJO/R95x).

Bone surface modification analyses (**Figures S5 to S7**) indicate that hominins had access to meat resources prior to any other carnivore. This is evidenced by cut marks located on the ventral side of ribs and on vertebrae bodies, which indicate evisceration activities (Figure S5a). The anatomical location of cut marks on long bones also reflects primary access. Cut marks are found mainly on the cranial, caudal, and medial aspects of humeri, but they also appear on the ends, maybe reflecting disarticulation. Moreover, as is the case at FLK Zinj, most of the marks observed on the radius cluster in hot zone 2 (cranial side), which is a very clear sign of filleting as has been observed experimentally [^1,17^](https://paperpile.com/c/LwZzJO/R95x+13RPD)). Furthermore, nine out of ten cut marks on femora appear on hot zones at DS (Figure S5b), indicating filleting, and tibiae are also characterized by a clearly higher frequency of cut marks in the hot zones at both DS and FLK Zinj (**Figure S5b**). This is especially revealing, because hind limbs have been observed to be defleshed by lions and leopards immediately after prey capture and evisceration [^1,66^](https://paperpile.com/c/LwZzJO/qZ9P+13RPD). The systematic presence of cut marks on hot zones at DS indicates that hominins were accessing carcasses before carnivores, as cut marks should never be found on hot zones after carnivore defleshing, especially with this proportion. It also means that hominins would have been removing complete muscles from the bones rather than flesh scraps. Additionally, the cut mark frequencies documented at DS coincide with cut mark frequencies documented experimentally in hominin-to-carnivore scenarios (Figure S5c), as do the combined bone surface modification frequencies (cut marks, percussion marks and tooth marks (Figure S6, Tables S7 and S8). Bone damage patterns also reveal some degree of carnivore damage that is not consistent with felid activity (Figure S7).

All the results of the presented taphonomic analyses reinforce previous interpretations about hominin behavior drawn from earlier taphonomic analyses of FLK *Zinj* and attest to the high explanatory power of multivariate and machine learning taphonomic approaches.

**
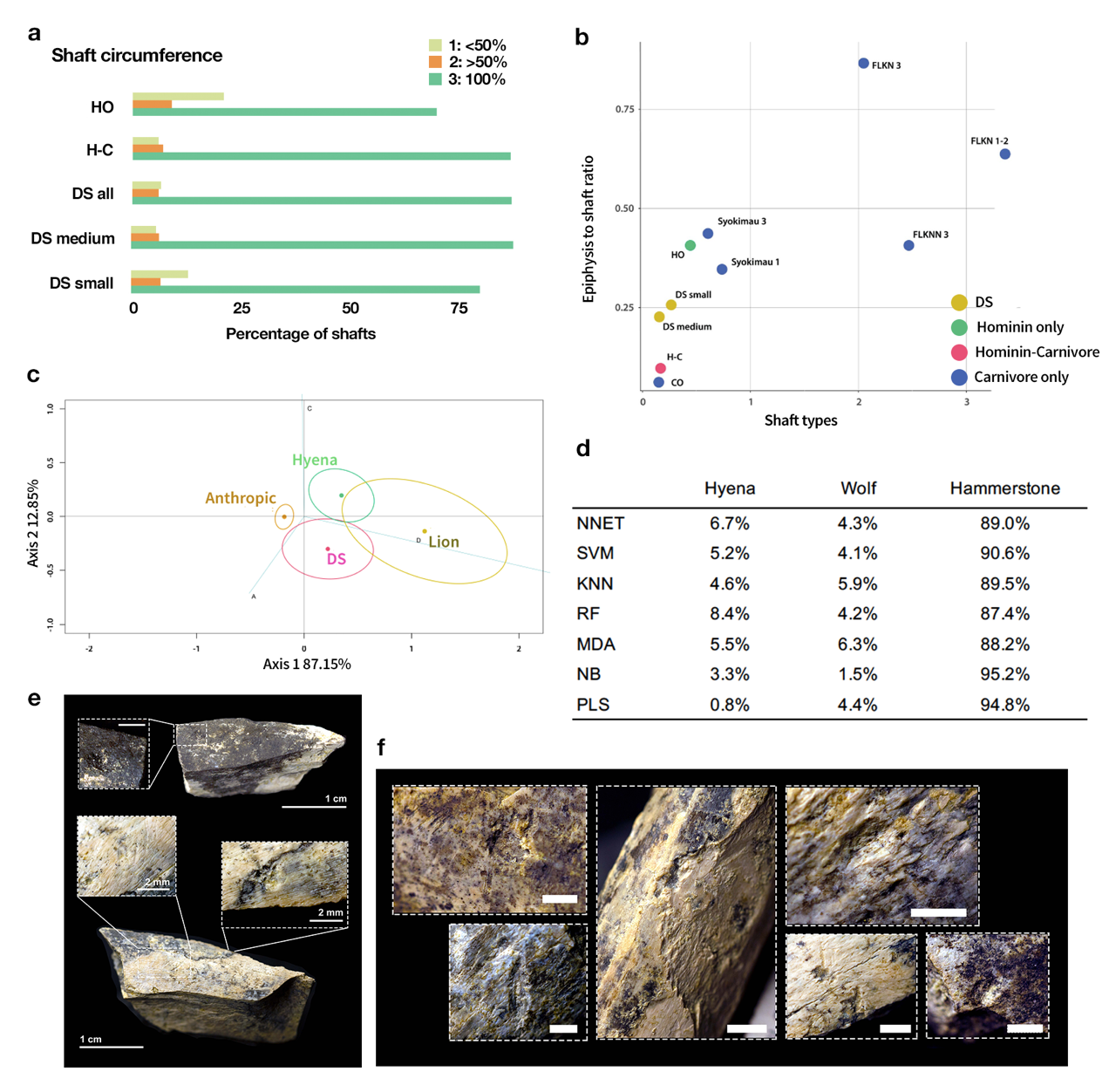
**

**Figure S4. Bone breakage patterns. a,** Bar chart showing the percentages of shaft circumference types of small and medium-sized carcasses at DS 22B compared to frequencies documented in experiments modeling primary access to carcasses by hominins and subsequent ravaging by hyenas (H-C) and hominin only (HO) [^67^](https://paperpile.com/c/LwZzJO/Q5Grr) . Comparative data on experimental carnivore-hominin scenarios were not available. Shaft type frequencies of small carcasses are similar to HO models, whereas shaft types of medium-sized carcasses coincide with H-C scenarios. **b,** Scatterplot combining shaft type proportions and epiphysis-to-shafts ratios from DS 22B, the HO and H-C experiments, Syokimau (hyena den) [^68^](https://paperpile.com/c/LwZzJO/ZqEhB), and several carnivore assemblages from Bed I and CO experiments [^1^](https://paperpile.com/c/LwZzJO/13RPD) following an approach presented by [^1^](https://paperpile.com/c/LwZzJO/13RPD). Unfortunately, data from carnivore to hominin scenarios are not available. The ratios from DS 22B fall closer to HO and H-C experiments than to carnivore assemblages. **c**, Two-dimensional solution (confidence regions for biplot of rows) explaining 100% of inertia of a bootstrapped correspondence analysis carried out on the notch types found on specimens from medium-sized carcasses, which points to a scenario in which bone fracturing was produced by both hominins and hyenas. The overlap with the lion sample is probably an artefact of small sample size, which produces a larger confidence ellipse. (see also Table S6). **d**, Classifications yielded by several machine learning analyses combining breakage plane angles and notch types (see approach by [^55^](https://paperpile.com/c/LwZzJO/BNkFv)) documented at DS 22B. NNET: Neural networks, SVM: Support Vector Machines, KNN: K Nearest Neighbor, RF: Random Forest, MDA: Mixture Discriminant Analysis, NB: Naïve Bayes, PLS: Partial Least Squares). Most breakage (~90%) was caused by hammerstone percussion, which means that hominin breakage activities seem to have been predominant, although hyenas contributed to some degree to bone breakage at the site. **e**, Two impact flakes with percussion pits on the cortical surface. **f**, Several examples of percussion marked long bone shafts.

Table S6. Frequencies of each type of notches on long limb bone fragments in DS (Level 22B). Percentages are in parentheses.

|  | Type A | Type B | Type C | Type D | Type E | Total |
| --- | --- | --- | --- | --- | --- | --- |
| Small carcasses | 7 (25) | 1 (3.5) | 4 (14.3) | 3 (10.7) | 13 (46.5) | 28 |
| Medium-sized carcasses | 21 (23.6) | 3 (3.4) | 6 (6.7) | 7 (7.9) | 52 (58.4) | 89 |
| Large carcasses | 2 (22.2) | 1 (11.1) | 0 | 0 | 6 (66.7) | 9 |
| All | 33 (25) | 4 (3) | 10 (7.6) | 10 (7.6) | 75 (56.9) | 132 |

**
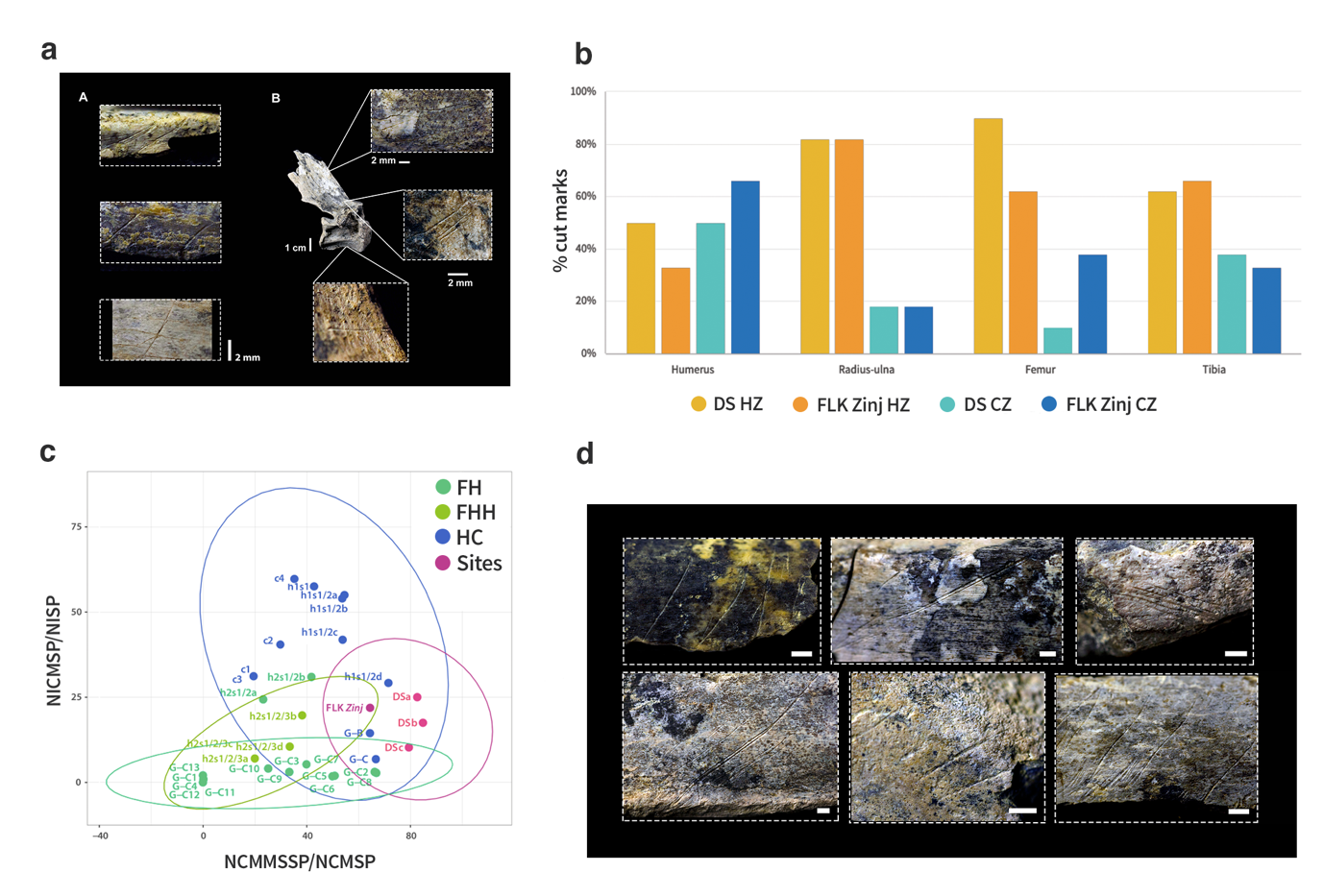
**

**Figure S5. Stone-tool butchery marks. a,** Stone tool cut marks located on the dorsal and ventral side of ribs and on vertebrae. **b**, Bar chart showing the distribution of cut marks on hot zones and cold zones on long bones in medium-sized carcasses at DS and FLK *Zinj*. Hot zones (HZ) on long bones are areas where there are no flesh scraps left after lion consumption, cold zones (CZ) are areas where flesh scraps are present after lions have consumed their prey [^1^](https://paperpile.com/c/LwZzJO/13RPD). At DS and FLK *Zinj*, cut marks are predominantly found on hot zones, **c**, Relationship between the number of identified cutmarked specimens / number of identified specimens ratio (NICMSP:NISP) and the number of identified cutmarked midshaft specimens / number of identified cut marked specimens ratio (NICMMSSP:NICMSP) for the medium-sized carcasses at DS and FLK *Zinj*, as well as for the experimental assemblages from the H-C (hominin to carnivore), F-H (felid-to-hominin), and F-H-H (felid-hominin-hyenid) models. DS and FLK *Zinj* fall within the H-C confidence ellipse. DSa refers to the subsample corrected for poor and moderate preservation as well as dry breakage (after [^69^](https://paperpile.com/c/LwZzJO/ysBBX)); DSb refers to the moderately preserved sample; DSc refers to the complete uncorrected sample. Data from experiments can be found in [^44–48,70–72^](https://paperpile.com/c/LwZzJO/drLUh+PPrE0+wmESE+TS4nH+Glbnk+RwaZy+AQ5EA+g7uOG)**. d**, Several examples of cut-marked long bone shafts.

**
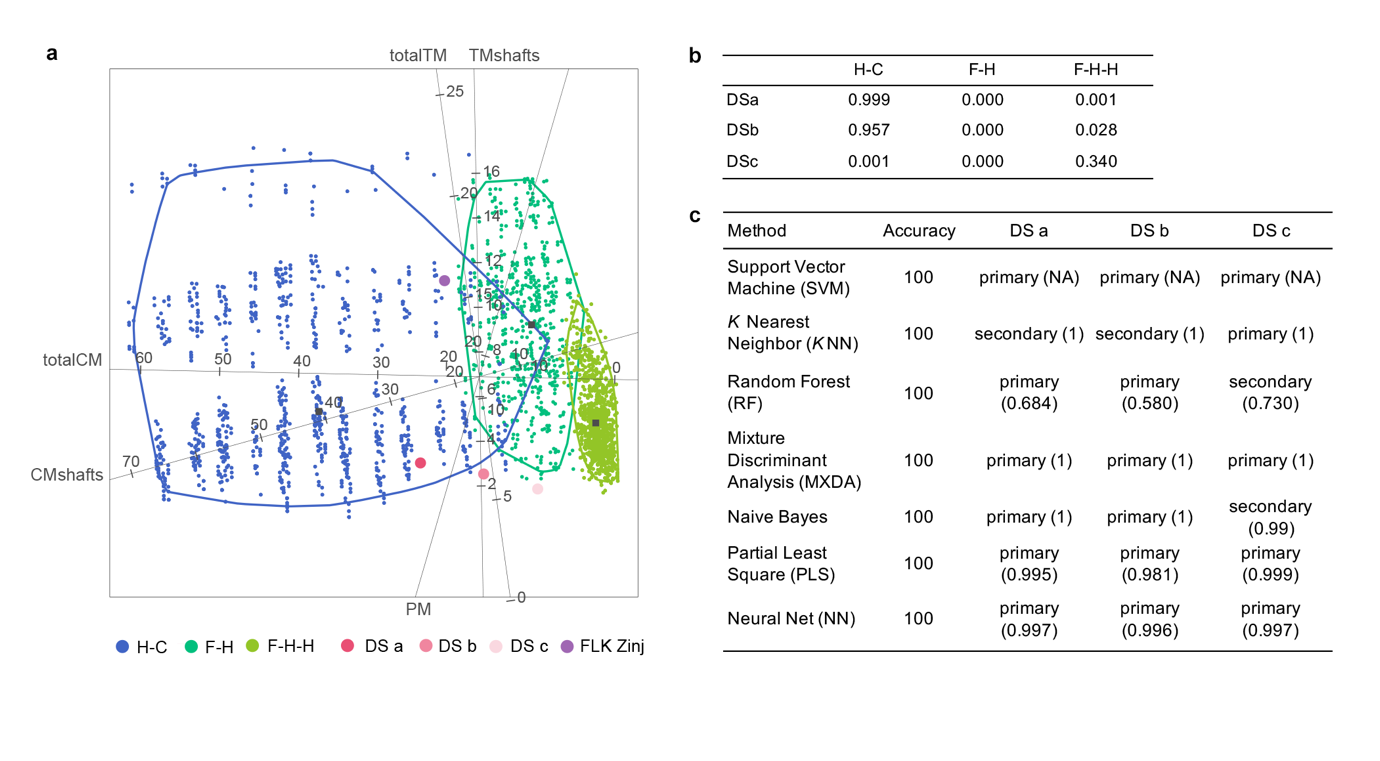
**

**Figure S6. Combined bone surface modifications multivariate and machine learning analyses. a,** Multiple discriminant analysis using a canonical variate approach on a bootstrapped sample of the experimental assemblages of the Hominin-Carnivore (dark blue alpha bag), Felid-Hominin (dark green alpha bag), Felid-Hominin-Hyenid (light green alpha bag) models. Experimental data from [^49^](https://paperpile.com/c/LwZzJO/FuLXo). The MDA correctly classified 91.9% of the experimental sample. Cut mark frequencies were more discriminatory than percussion marks and tooth marks. The well-preserved DS assemblage appears within the 95% confidence alpha bag of the Hominin-Carnivore model and is placed, as is the case for FLK *Zinj*, towards the right elongation of the Hominin-Carnivore alpha bag, which includes experiments reproducing bulk flesh removal. **b,** Predictions of the model. Classifications of DS samples in the three experimental models. The well-preserved DS sample is classified as primary access with a probability of 99%, DSb is classified as primary access with a probability of 96% and the uncorrected sample is classified with the Felid-Hominin experiments with a probability of 34%. **c,** Accuracy percentages of correct classification of each statistical algorithm and classification into primary or secondary access of DS. All methods yield 100% accuracy. Classifications were nearly always higher than 95%. Overall, DS is classified as primary access more than 80% of the times.

Table S7. Mean percentages of cut marked specimens in each skeletal section per NISP (A) and per total number of cut marked specimens (B) for small and medium-sized carcasses in the DS subassemblages, and in FLK Zinj 22.

A)

| Size 1-2 |  | **ULB** | **ILB** | **LLB** | **MSH** | **Ends** | **Total** |
| --- | --- | --- | --- | --- | --- | --- | --- |
| DS a | mean | 12.5 | 15.8 | 0 | 13.3 | 7.1 | 11.7 |
|  |  |  |  |  |  |  |  |
| DS b | mean | 10.7 | 12 | 0 | 10.7 | 4.7 | 9.03 |
|  |  |  |  |  |  |  |  |
| DS c | mean | 6.49 | 7.14 | 3.23 | 6.92 | 4.17 | 6.18 |
|  |  |  |  |  |  |  |  |
| FLK Zinj | mean | 20 | 19.7 | 6.2 | 11.9 | 21.8 | 14.9 |
|  |  |  |  |  |  |  |  |
|  | | | | | | | |
| Size 3-4 |  | **ULB** | **ILB** | **LLB** | **MSH** | **Ends** | **Total** |
| DS a | mean | 24.2 | 30.8 | 18.2 | 27.3 | 17.9 | 25 |
|  |  |  |  |  |  |  |  |
| DS b | mean | 13.8 | 21.7 | 18.6 | 18.6 | 35.2 | 17.5 |
|  |  |  |  |  |  |  |  |
| DS c | mean | 8.59 | 12.16 | 9.71 | 10.3 | 10.17 | 10.26 |
|  |  |  |  |  |  |  |  |
| FLK Zinj | mean | 25.5 | 23.1 | 12.3 | 17.2 | 46.6 | 21.9 |
|  |  |  |  |  |  |  |  |

Table S8. Discriminant coefficient scores for the first three functions of the MXDA test

|  | Function 1 | Function 2 | Function 3 |
| --- | --- | --- | --- |
| Total CM | 0.037373360 | 0.0243881326 | 0.028237409 |
| CM shafts | 0.020723473 | 0.0507520323 | 0.001495505 |
| PM shafts | 0.002731512 | 0.0009340008 | 0.072427193 |
| Total TM | 0.011428745 | 0.0058899413 | 0.012719631 |
| TM shafts | 0.002328794 | 0.0022947592 | 0.012956717 |

**
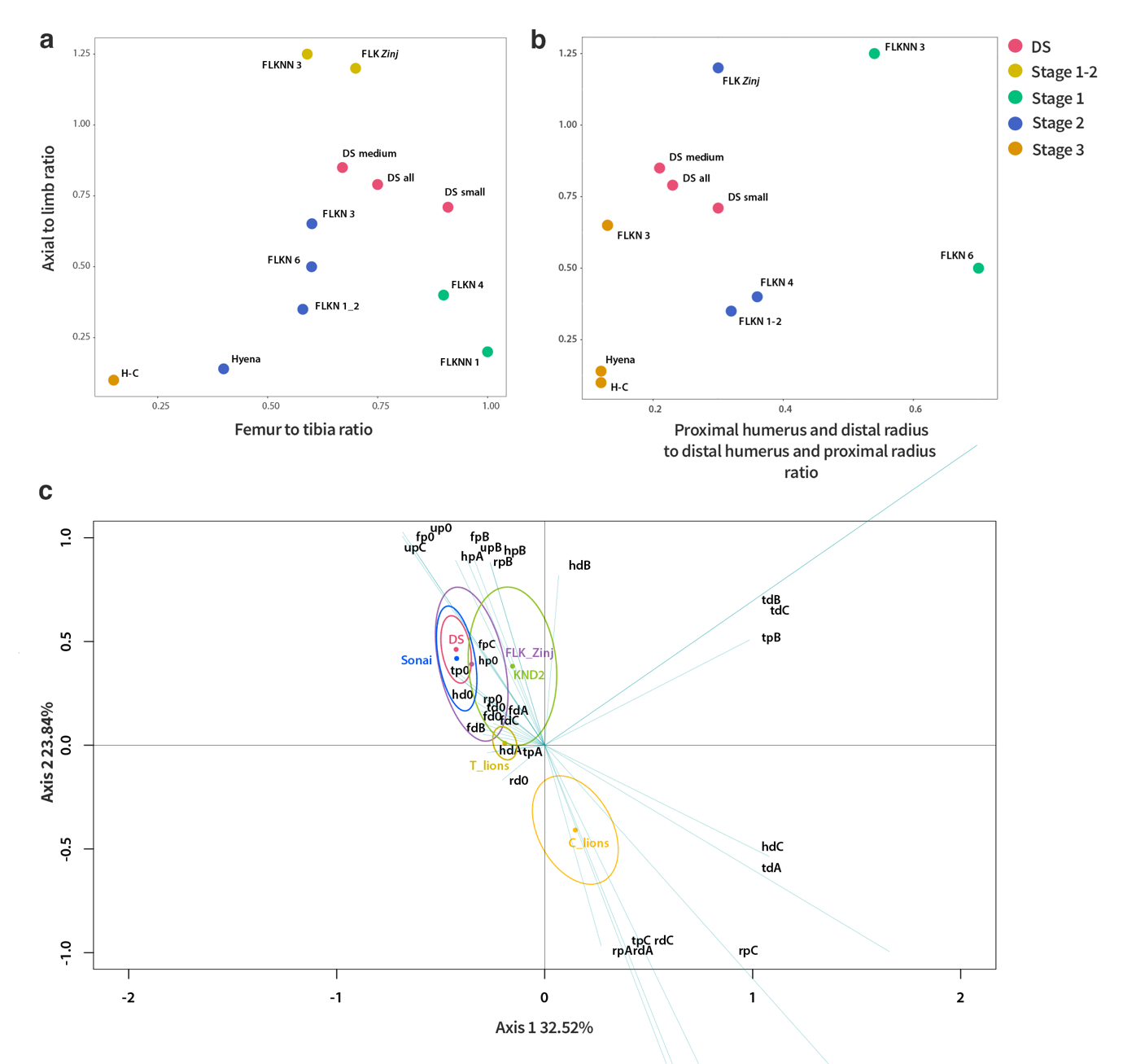
**

**Figure S7. Evidence of ravaging by durophagous carnivores. a**, Degree of ravaging documented at DS 22B compared to several other bone assemblages from the Olduvai Bed I sites using the femur to tibia ratio and **b**, the proximal humerus and distal radius to distal humerus and proximal radius ratio. DS 22B falls between stages 1 and 2, and between 2 and 3 respectively, which suggests slightly higher degrees of ravaging than at FLK *Zinj* (see [^1^](https://paperpile.com/c/LwZzJO/13RPD)). Carnivore competition at DS could have been moderate. However, DS seems to have been located in a closed-vegetation setting ([^40^](https://paperpile.com/c/LwZzJO/QLEcL). These areas would probably still have remained the lowest competition settings on the paleolandscape. **c**, Biplot of columns of the bootstrapped correspondence analysis carried out on taphotypes (bone damage patterns on long bones, see [^56^](https://paperpile.com/c/LwZzJO/nvtv4) showing the relationship between the referential carnivore and anthropic CSII taphotypes and the ones documented at DS 22B considering all long bones. The bone damage pattern documented at DS overlaps with the Sonai sample (anthropogenic) and also falls inside the FLK *Zinj* confidence ellipse. Importantly, no overlap occurs between the DS sample and the samples modified by felids, which rules out that felids could have accumulated or modified carcasses at DS

**6. References**

1. [Domínguez-Rodrigo, M., Egido, R. B. & Egeland, C. P. Deconstructing Olduvai: A Taphonomic Study of the Bed I Sites. *Vertebrate Paleobiology and Paleoanthropology* (2007) doi:](http://paperpile.com/b/LwZzJO/13RPD)[10.1007/978-1-4020-6152-3](http://dx.doi.org/10.1007/978-1-4020-6152-3)[.](http://paperpile.com/b/LwZzJO/13RPD)

2. [Plummer, T. W. *et al.* The Environmental Context of Oldowan Hominin Activities at Kanjera South, Kenya. in *Interdisciplinary Approaches to the Oldowan* (eds. Hovers, E. & Braun, D. R.) 149–160 (Springer Netherlands, 2009).](http://paperpile.com/b/LwZzJO/kjugH)

3. [Plummer, T. & Bishop, L. Oldowan hominin behavior and ecology at Kanjera South,Kenya. *J. Anthropol. Sci.* **94**, 29–40 (2016).](http://paperpile.com/b/LwZzJO/YZXBM)

4. [Oliver, J. S., Plummer, T. W., Hertel, F. & Bishop, L. C. Bovid mortality patterns from Kanjera South, Homa Peninsula, Kenya and FLK-Zinj, Olduvai Gorge, Tanzania: Evidence for habitat mediated variability in Oldowan hominin hunting and scavenging behavior. *Journal of Human Evolution* vol. 131 61–75 (2019).](http://paperpile.com/b/LwZzJO/tWRZD)

5. [Bunn, H. *et al.* FxJj50: An early Pleistocene site in northern Kenya. *World Archaeol.* **12**, 109–136 (1980).](http://paperpile.com/b/LwZzJO/coMLZ)

6. [Braun, D. R., Rogers, M. J., Harris, J. W. & Walker, S. J. Quantifying Variation in Landscape-Scale Behaviors: The Oldowan from Koobi Fora. *New Perspectives on Old Stones* 167–182 (2010) doi:](http://paperpile.com/b/LwZzJO/o8suK)[10.1007/978-1-4419-6861-6_7](http://dx.doi.org/10.1007/978-1-4419-6861-6_7)[.](http://paperpile.com/b/LwZzJO/o8suK)

7. [Bunn, H. T. *et al.* Systematic Butchery by Plio/Pleistocene Hominids at Olduvai Gorge, Tanzania [and Comments and Reply]. *Curr. Anthropol.* **27**, 431–452 (1986).](http://paperpile.com/b/LwZzJO/GZxk8)

8. [Oliver, J. S. Estimates of hominid and carnivore involvement in the FLK Zinjanthropus fossil assemblage: some socioecological implications. *J. Hum. Evol.* **27**, 267–294 (1994).](http://paperpile.com/b/LwZzJO/iR84r)

9. [Blumenschine, R. J. Hominid carnivory and foraging strategies, and the socio-economic function of early archaeological sites. *Philos. Trans. R. Soc. Lond. B Biol. Sci.* **334**, 211–9; discussion 219–21 (1991).](http://paperpile.com/b/LwZzJO/hfXu8)

10. [Bunn, H. T. & Pickering, T. R. Bovid mortality profiles in paleoecological context falsify hypotheses of endurance running–hunting and passive scavenging by early Pleistocene hominins. *Quat. Res.* **74**, 395–404 (2010).](http://paperpile.com/b/LwZzJO/AUl7P)

11. [Parkinson, J. A. *A GIS image analysis approach to documenting Oldowan hominin carcass acquisition: Evidence from Kanjera South, FLK Zinj, and neotaphonomic models of carnivore bone destruction*. (City University of New York, 2013).](http://paperpile.com/b/LwZzJO/IhOAc)

12. [Domínguez-Rodrigo, M. Taphonomy in early African archaeological sites: Questioning some bone surface modification models for inferring fossil hominin and carnivore feeding interactions. *J. Afr. Earth. Sci.* **108**, 42–46 (2015).](http://paperpile.com/b/LwZzJO/UUlPm)

13. [Domínguez-Rodrigo, M. & Cobo-Sánchez, L. A spatial analysis of stone tools and fossil bones at FLK Zinj 22 and PTK I (Bed I, Olduvai Gorge, Tanzania) and its bearing on the social organization of early humans. *Palaeogeogr. Palaeoclimatol. Palaeoecol.* **488**, 21–34 (2017).](http://paperpile.com/b/LwZzJO/SHJfp)

14. [Domínguez-Rodrigo, M., Cobo-Sánchez, L., Aramendi, J. & Gidna, A. The meta-group social network of early humans: A temporal–spatial assessment of group size at FLK Zinj (Olduvai Gorge, Tanzania). *J. Hum. Evol.* **127**, 54–66 (2019).](http://paperpile.com/b/LwZzJO/0qlkX)

15. [Domínguez-Rodrigo, M. *et al.* Constraining time and ecology on the Zinj paleolandscape: Microwear and mesowear analyses of the archaeofaunal remains of FLK Zinj and DS (Bed I), compared to FLK North (Bed I) and BK (Bed II) at Olduvai Gorge (Tanzania). *Quat. Int.* **526**, 4–14 (2019).](http://paperpile.com/b/LwZzJO/7FqA5)

16. [Domínguez-Rodrigo, M. & Pickering, T. R. Early hominid hunting and scavenging: A zooarcheological review. *Evolutionary Anthropology: Issues, News, and Reviews* vol. 12 275–282 (2003).](http://paperpile.com/b/LwZzJO/hYUL)

17. [Domínguez-Rodrigo, M., Barba, R. & Egeland, C. P. *Deconstructing Olduvai: A Taphonomic Study of the Bed I Sites*. (Springer Science & Business Media, 2007).](http://paperpile.com/b/LwZzJO/R95x)

18. [Domínguez-Rodrigo, M. & Pickering, T. R. The meat of the matter: an evolutionary perspective on human carnivory. *Azania: Archaeological Research in Africa* vol. 52 4–32 (2017).](http://paperpile.com/b/LwZzJO/5kAL)

19. [Domínguez-Rodrigo, M. *Stone Tools and Fossil Bones: Debates in the Archaeology of Human Origins*. (Cambridge University Press, 2012).](http://paperpile.com/b/LwZzJO/mfgj)

20. [Leakey, M. D. *Olduvai Gorge: Volume 3, Excavations in Beds I and II, 1960-1963*. (Cambridge University Press, 1971).](http://paperpile.com/b/LwZzJO/EFcHa)

21. [Parkinson, J. A. Revisiting the hunting-versus-scavenging debate at FLK Zinj: A GIS spatial analysis of bone surface modifications produced by hominins and carnivores in the FLK 22 assemblage, Olduvai Gorge, Tanzania. *Palaeogeography, Palaeoclimatology, Palaeoecology* vol. 511 29–51 (2018).](http://paperpile.com/b/LwZzJO/7QhYP)

22. [Domínguez-Rodrigo, M. & Pickering, T. R. Earliest modern human-like hand bone from a new> 1.84-million-year-old site at Olduvai in Tanzania. *Nature* (2015).](http://paperpile.com/b/LwZzJO/jvrps)

23. [Villa, P. & Lenoir, M. Hunting and Hunting Weapons of the Lower and Middle Paleolithic of Europe. in *The Evolution of Hominin Diets: Integrating Approaches to the Study of Palaeolithic Subsistence* (eds. Hublin, J.-J. & Richards, M. P.) 59–85 (Springer Netherlands, 2009).](http://paperpile.com/b/LwZzJO/FB08M)

24. [Richards, M. P. & Schmitz, R. W. Isotope evidence for the diet of the Neanderthal type specimen. *Antiquity* vol. 82 553–559 (2008).](http://paperpile.com/b/LwZzJO/9PBlN)

25. [Berger, T. D. & Trinkaus, E. Patterns of trauma among the Neandertals. *J. Archaeol. Sci.* (1995).](http://paperpile.com/b/LwZzJO/XjUrA)

26. [Saladié, P. *et al.* The role of carnivores and their relationship to hominin settlements in the TD6-2 level from Gran Dolina (Sierra de Atapuerca, Spain). *Quat. Sci. Rev.* **93**, 47–66 (2014).](http://paperpile.com/b/LwZzJO/V6Mvf)

27. [Rodriguez-Hidalgo, A. *et al.* Human predatory behavior and the social implications of communal hunting based on evidence from the TD10. 2 bison bone bed at Gran Dolina (Atapuerca, Spain). *J. Hum. Evol.* **105**, 89–122 (2017).](http://paperpile.com/b/LwZzJO/itEZc)

28. [Lupo, K. D. On early hominin meat eating and carcass acquisition strategies. *Stone Tools and Fossil Bones* 115–151 doi:](http://paperpile.com/b/LwZzJO/Hc76i)[10.1017/cbo9781139149327.006](http://dx.doi.org/10.1017/cbo9781139149327.006)[.](http://paperpile.com/b/LwZzJO/Hc76i)

29. [Isaac, G. The Food-sharing Behavior of Protohuman Hominids. *Scientific American* vol. 238 90–108 (1978).](http://paperpile.com/b/LwZzJO/DuBIG)

30. [Pickering, T. R. & Bunn, H. T. Meat foraging by Pleistocene African hominins. *Stone Tools and Fossil Bones* 152–173 doi:](http://paperpile.com/b/LwZzJO/uRWyW)[10.1017/cbo9781139149327.007](http://dx.doi.org/10.1017/cbo9781139149327.007)[.](http://paperpile.com/b/LwZzJO/uRWyW)

31. [Uribelarrea, D. *et al.* Geo-archaeological and geometrically corrected reconstruction of the 1.84 Ma FLK Zinj paleolandscape at Olduvai Gorge, Tanzania. *Quat. Int.* **322-323**, 7–31 (2014).](http://paperpile.com/b/LwZzJO/pmrXP)

32. [Aiello, L. C. & Wheeler, P. The Expensive-Tissue Hypothesis: The Brain and the Digestive System in Human and Primate Evolution. *Curr. Anthropol.* **36**, 199–221 (1995).](http://paperpile.com/b/LwZzJO/JxP1V)

33. [Domínguez-Rodrigo, M. *et al.* Spatial simulation and modelling of the early Pleistocene site of DS (Bed I, Olduvai Gorge, Tanzania): a powerful tool for predicting potential archaeological information from unexcavated areas. *Boreas* **46**, 805–815 (2017).](http://paperpile.com/b/LwZzJO/eBRCD)

34. [Diez-Martín, F. *et al.* Tracing the spatial imprint of Oldowan technological behaviors: A view from DS (Bed I, Olduvai Gorge, Tanzania). *PLoS One* **16**, e0254603 (2021).](http://paperpile.com/b/LwZzJO/HBUW)

35. [Walter, R. C., Manega, P. C., Hay, R. L., Drake, R. E. & Curtis, G. H. Laser-fusion 40Ar/39Ar dating of Bed I, Olduvai Gorge, Tanzania. *Nature* **354**, 145–149 (1991).](http://paperpile.com/b/LwZzJO/a2TUi)

36. [Walter, R. C., Manega, P. C. & Hay, R. L. Tephrochronology of Bed I, Olduvai Gorge: An application of laser-fusion 40Ar39Ar dating to calibrating biological and climatic change. *Quat. Int.* **13-14**, 37–46 (1992).](http://paperpile.com/b/LwZzJO/r2qlF)

37. [Manega, P. C. Geochronology, geochemistry and isotopic study of the Plio-Pleistocene hominid sites and the Ngorongoro Volcanic Highland in northern Tanzania. (1994).](http://paperpile.com/b/LwZzJO/Npi4z)

38. [Blumenschine, R. J. *et al.* Late Pliocene Homo and hominid land use from Western Olduvai Gorge, Tanzania. *Science* **299**, 1217–1221 (2003).](http://paperpile.com/b/LwZzJO/XpQab)

39. [Martín-Perea, D. M. *et al.* Mineral assemblages and low energy sedimentary processes in the FLK-Zinj, DS, PTK and AMK complex palaeolandscape (Olduvai Gorge, Tanzania). *Quat. Int.* **526**, 15–25 (2019).](http://paperpile.com/b/LwZzJO/8IgC0)

40. [Arráiz, H. *et al.* The FLK Zinj paleolandscape: Reconstruction of a 1.84Ma wooded habitat in the FLK Zinj-AMK-PTK-DS archaeological complex, Middle Bed I (Olduvai Gorge, Tanzania). *Palaeogeogr. Palaeoclimatol. Palaeoecol.* **488**, 9–20 (2017).](http://paperpile.com/b/LwZzJO/QLEcL)

41. [Hay, R. L. *Geology of the Olduvai Gorge: A Study of Sedimentation in a Semiarid Basin*. (University of California Press, 1976).](http://paperpile.com/b/LwZzJO/MsdIO)

42. [Capaldo, S. D. & Blumenschine, R. J. A Quantitative Diagnosis of Notches Made by Hammerstone Percussion and Carnivore Gnawing on Bovid Long Bones. *American Antiquity* vol. 59 724–748 (1994).](http://paperpile.com/b/LwZzJO/AtbUA)

43. [Pickering, T. R. & Egeland, C. P. Experimental patterns of hammerstone percussion damage on bones: implications for inferences of carcass processing by humans. *Journal of Archaeological Science* vol. 33 459–469 (2006).](http://paperpile.com/b/LwZzJO/0wvFA)

44. [Blumenschine, R. J. An experimental model of the timing of hominid and carnivore influence on archaeological bone assemblages. *Journal of Archaeological Science* vol. 15 483–502 (1988).](http://paperpile.com/b/LwZzJO/PPrE0)

45. [Blumenschine, R. J. Percussion marks, tooth marks, and experimental determinations of the timing of hominid and carnivore access to long bones at FLK Zinjanthropus, Olduvai Gorge, Tanzania. *Journal of Human Evolution* vol. 29 21–51 (1995).](http://paperpile.com/b/LwZzJO/wmESE)

46. [Capaldo, S. D. *Inferring Hominid and Carnivore Behavior from Dual-patterned Archaeofaunal Assemblages*. (Rutgers University, 1995).](http://paperpile.com/b/LwZzJO/TS4nH)

47. [Domínguez-Rodrigo, M. Testing meat-eating in early hominids: an analysis of butchery marks on defleshed carcases. *Hum. Evol.* **12**, 169–182 (1997).](http://paperpile.com/b/LwZzJO/drLUh)

48. Domínguez-[Rodrigo, M. D. & Barba, R. A study of cut marks on small-sized carcasses and its application to the study of cut-marked bones from small mammals at the FLK Zinj site. *Journal of taphonomy* **3**, 121–134 (2005).](http://paperpile.com/b/LwZzJO/g7uOG)

49. [Domínguez-Rodrigo, M., Bunn, H. T. & Yravedra, J. A critical re-evaluation of bone surface modification models for inferring fossil hominin and carnivore interactions through a multivariate approach: application to the FLK Zinj archaeofaunal assemblage (Olduvai Gorge, Tanzania). *Quat. Int.* **322**, 32–43 (2014).](http://paperpile.com/b/LwZzJO/FuLXo)

50. [Domínguez-Rodrigo, M. *et al.* Fluvial spatial taphonomy: a new method for the study of post-depositional processes. *Archaeological and Anthropological Sciences* vol. 10 1769–1789 (2018).](http://paperpile.com/b/LwZzJO/ttAJq)

51. [Domínguez-Rodrigo, M., Baquedano, E., Barba, R., Uribelarrea, D. & Gidna, A. The river that never was: Fluvial taphonomy at Olduvai Bed I and II sites and its bearing on early human behavior. *Quat. Int.* **526**, 26–38 (2019).](http://paperpile.com/b/LwZzJO/GIdfG)

52. [Arriaza, M. C. & Domínguez-Rodrigo, M. When felids and hominins ruled at Olduvai Gorge: A machine learning analysis of the skeletal profiles of the non-anthropogenic Bed I sites. *Quat. Sci. Rev.* **139**, 43–52 (2016).](http://paperpile.com/b/LwZzJO/r9UKs)

53. [Domínguez-Rodrigo, M. & Baquedano, E. Distinguishing butchery cut marks from crocodile bite marks through machine learning methods. *Sci. Rep.* **8**, 5786 (2018).](http://paperpile.com/b/LwZzJO/CVqrW)

54. [Domínguez-Rodrigo, M. Successful classification of experimental bone surface modifications (BSM) through machine learning algorithms: a solution to the controversial use of BSM in …. *Archaeol. Anthropol. Sci.* (2019).](http://paperpile.com/b/LwZzJO/OkkXV)

55. [Moclán, A., Domínguez-Rodrigo, M. & Yravedra, J. Classifying agency in bone breakage: an experimental analysis of fracture planes to differentiate between hominin and carnivore dynamic and static loading using machine learning (ML) algorithms. *Archaeol. Anthropol. Sci.* **11**, 4663–4680 (2019).](http://paperpile.com/b/LwZzJO/BNkFv)

56. [Domínguez-Rodrigo, M. *et al.* A new methodological approach to the taphonomic study of paleontological and archaeological faunal assemblages: a preliminary case study from Olduvai Gorge (Tanzania). *J. Archaeol. Sci.* **59**, 35–53 (2015).](http://paperpile.com/b/LwZzJO/nvtv4)

57. [Behrensmeyer, A. K. Taphonomic and ecologic information from bone weathering. *Paleobiology* vol. 4 150–162 (1978).](http://paperpile.com/b/LwZzJO/227r)

58. [Lê, S., Josse, J. & Husson, F. FactoMineR: AnRPackage for Multivariate Analysis. *Journal of Statistical Software* vol. 25 (2008).](http://paperpile.com/b/LwZzJO/XTdX)

59. Grohmann, C. H., & Campanha, G. A. (2010, December). OpenStereo: open source, cross-platform software for structural geology analysis. In *AGU Fall Meeting abstracts* (Vol. 2010, pp. IN31C-06).

60. [Woodcock, N. H. Specification of fabric shapes using an eigenvalue method. *Geological Society of America Bulletin* vol. 88 1231 (1977).](http://paperpile.com/b/LwZzJO/jJrC)

61. [Benn, D. I. Fabric Shape and the Interpretation of Sedimentary Fabric Data. *SEPM Journal of Sedimentary Research* vol. 64A (1994).](http://paperpile.com/b/LwZzJO/cjKc)

62. [Pewsey, A., Neuhäuser, M. & Ruxton, G. D. *Circular Statistics in R*. (OUP Oxford, 2013).](http://paperpile.com/b/LwZzJO/SGtw)

63. [Bunn, H. T. Animal Bones and Archeological Inference. *Science* vol. 215 494–495 (1982).](http://paperpile.com/b/LwZzJO/X433e)

64. [Lam, Y. M., Chen, X. & Pearson, O. M. Intertaxonomic Variability in Patterns of Bone Density and the Differential Representation of Bovid, Cervid, and Equid Elements in the Archaeological Record. *American Antiquity* vol. 64 343–362 (1999).](http://paperpile.com/b/LwZzJO/zKT5)

65. [Lyman, R. L. *The Taphonomy of Vertebrate Archaeofaunas: Bone Density and Differential Survivorship of Fossil Classes*.](http://paperpile.com/b/LwZzJO/1nQk)

66. [Blumenschine, R. J. *Early Hominid Scavenging Opportunities: Implications of Carcass Availability in the Serengeti and Ngorongoro Ecosystems*. (British Archaeological Reports Limited, 1986).](http://paperpile.com/b/LwZzJO/qZ9P)

67. [Marean, C. W., Rodrigo, M. D. & Pickering, T. R. Skeletal element equifinality in zooarchaeology begins with method: The evolution and status of the ‘shaft critique’. *Journal of taphonomy* **2**, 69–98 (2004).](http://paperpile.com/b/LwZzJO/Q5Grr)

68. [Egeland, A. G., Egeland, C. P. & Bunn, H. T. Taphonomic analysis of a modern spotted hyena (Crocuta crocuta) den from Nairobi, Kenya. *Journal of Taphonomy* **6**, 275–299 (2008).](http://paperpile.com/b/LwZzJO/ZqEhB)

69. [Pickering, T. R., Egeland, C. P., Domínguez-Rodrigo, M., Brain, C. K. & Schnell, A. G. Testing the ‘shift in the balance of power’ hypothesis at Swartkrans, South Africa: Hominid cave use and subsistence behavior in the Early Pleistocene. *Journal of Anthropological Archaeology* **27**, 30–45 (2008).](http://paperpile.com/b/LwZzJO/ysBBX)

70. [Capaldo, S. D. Experimental determinations of carcass processing by Plio-Pleistocene hominids and carnivores at FLK 22 (Zinjanthropus). Olduvai Gorge, Tanzania. *J. Hum. Evol.* **33**, 555–597 (1997).](http://paperpile.com/b/LwZzJO/Glbnk)

71. [Pante, M. C., Blumenschine, R. J., Capaldo, S. D. & Scott, R. S. Validation of bone surface modification models for inferring fossil hominin and carnivore feeding interactions, with reapplication to FLK 22, Olduvai Gorge, Tanzania. *J. Hum. Evol.* **63**, 395–407 (2012).](http://paperpile.com/b/LwZzJO/RwaZy)

72. [Gidna, A. O., Kisui, B., Mabulla, A., Musiba, C. & Domínguez-Rodrigo, M. An ecological neo-taphonomic study of carcass consumption by lions in Tarangire National Park (Tanzania) and its relevance for human evolutionary biology. *Quaternary International* vols 322-323 167–180 (2014).](http://paperpile.com/b/LwZzJO/AQ5EA)

**7. Major indicators of the experimental transfer models**

PITS

RESNET 50 (without and with Dropout)

val_loss: 0.0962 (0.0891)- val_accuracy: 0.9565

[[14 0]

[ 1 8]]

precision recall f1-score support

0 0.93 1.00 0.97 14

1 1.00 0.89 0.94 9

accuracy 0.96 23

macro avg 0.97 0.94 0.95 23

weighted avg 0.96 0.96 0.96 23

auc 0.9444444444444444

-------------------------------------------------------------

VGG19 (without Dropout)

val_loss: 0.0491 - val_accuracy: 1.0000

[[14 0]

[ 0 9]]

precision recall f1-score support

0 1.00 1.00 1.00 14

1 1.00 1.00 1.00 9

accuracy 1.00 23

macro avg 1.00 1.00 1.00 23

weighted avg 1.00 1.00 1.00 23

auc= 1.0

--------------------------------------------------------------

VGG19 (with Dropout)

val_loss: 0.0901 - val_accuracy: 0.9565

[[14 0]

[ 1 8]]

precision recall f1-score support

0 0.93 1.00 0.97 14

1 1.00 0.89 0.94 9

accuracy 0.96 23

macro avg 0.97 0.94 0.95 23

weighted avg 0.96 0.96 0.96 23

auc 0.9444444444444444

\\\\\\\\\\\\\\\\\\\\\\\\\\\\\\\\\\\\\\\\\\\\\\\\\\\\\\\\\\\\\\\\\\\\\\\\\\\\\\\\\\\\\\\\\\\\\\\\\\\\\\\\\\\\\\\\\\\\\\\\\\\\\\\\\\\\\\\\\\\\\\\\\\\

SCORES

VGG19 (without Dropout)

val_loss: 0.2943 - val_accuracy: 0.8841

[[19 1]

[ 7 42]]

precision recall f1-score support

0 0.73 0.95 0.83 20

1 0.98 0.86 0.91 49

accuracy 0.88 69

macro avg 0.85 0.90 0.87 69

weighted avg 0.91 0.88 0.89 69

auc 0.9035714285714286

------------------------------------------------

VGG19 (with Dropout)

val_loss: 0.3882 - val_accuracy: 0.8551

[[17 3]

[ 7 42]]

precision recall f1-score support

0 0.71 0.85 0.77 20

1 0.93 0.86 0.89 49

accuracy 0.86 69

macro avg 0.82 0.85 0.83 69

weighted avg 0.87 0.86 0.86 69

auc 0.8535714285714285

--------------------------------------------------------------

VGG19 (with Dropout and Early Stopping)

val_loss: 0.6431 - val_accuracy: 0.6667

[[19 1]

[22 27]]

precision recall f1-score support

0 0.46 0.95 0.62 20

1 0.96 0.55 0.70 49

accuracy 0.67 69

macro avg 0.71 0.75 0.66 69

weighted avg 0.82 0.67 0.68 69

AUC: 0.7505102040816326

------------------------------------------------

RESNET (without dropout)

val_loss: 0.1168 - val_accuracy: 0.9565

[[18 2]

[ 1 48]]

precision recall f1-score support

0 0.95 0.90 0.92 20

1 0.96 0.98 0.97 49

accuracy 0.96 69

macro avg 0.95 0.94 0.95 69

weighted avg 0.96 0.96 0.96 69

auc 0.939795918367347

--------------------------------------------------

RESNET (with dropout)

val_loss: 0.1983 - val_accuracy: 0.9420

[[17 3]

[ 1 48]]

precision recall f1-score support

0 0.95 0.90 0.92 20

1 0.96 0.98 0.97 49

accuracy 0.96 69

macro avg 0.95 0.94 0.95 69

weighted avg 0.96 0.96 0.96 69

0.9147959183673469

------------------------------------------------------

RESNET (with dropout and early stopping)

val_loss: 0.3772 - val_accuracy: 0.8406

[[17 3]

[ 8 41]]

precision recall f1-score support

0 0.68 0.85 0.76 20

1 0.93 0.84 0.88 49

accuracy 0.84 69

macro avg 0.81 0.84 0.82 69

weighted avg 0.86 0.84 0.85 69

AUC: 0.8433673469387756
